# Development and Properties of Disposable Plates Made of Cellulosic Pulp from Mango Agro-Industrial Waste

**DOI:** 10.3390/polym17202757

**Published:** 2025-10-15

**Authors:** Maribel García-Mahecha, Herlinda Soto-Valdez, María Guadalupe Lomelí-Ramírez, Hilda Palacios-Juárez, José Anzaldo-Hernández, Tomás Jesús Madera-Santana, Citlali Colín-Chávez, Elizabeth Peralta, Rafael Auras, Elizabeth Carvajal-Millan

**Affiliations:** 1 Coordinación de Tecnología de Alimentos de Origen Vegetal, Centro de Investigación en Alimentación y Desarrollo, A.C. (CIAD), Hermosillo 83304, Sonora, Mexico; maribel.garciam@hotmail.com (M.G.-M.); madera@ciad.mx (T.J.M.-S.); eperalta@ciad.mx (E.P.); 2 Departamento de Madera, Celulosa y Papel del Centro Universitario de Ciencias Exactas e Ingenierías, Universidad de Guadalajara, Km 15.5 Carretera Guadalajara-Nogales, Zapopan 45220, Jalisco, Mexico; maria.lramirez@academicos.udg.mx (M.G.L.-R.); hilda.palacios@academicos.udg.mx (H.P.-J.); jose.anzaldo@academicos.udg.mx (J.A.-H.); 3 Centro de Innovación y Desarrollo Agroalimentario de Michoacán, A.C. (CIDAM), Morelia 58341, Michoacán, Mexico; citlali.colin@ciad.mx; 4 School of Packaging, Michigan State University, East Lansing, MI 48424, USA; aurasraf@msu.edu; 5 Coordinación de Tecnología de Alimentos de Origen Animal, Centro de Investigación para Alimentación y Desarrollo, A.C., (CIAD), Hermosillo 83304, Sonora, Mexico; ecarvajal@ciad.mx

**Keywords:** mango cellulosic pulp, seed tegument, disposable plates, water resistance, biodegradation

## Abstract

Recent studies have shown that using conventional plastics to produce disposable tableware significantly impacts the environment. Alternatively, using cellulosic pulp from harnessing agro-industrial wastes, such as mango, provides a unique opportunity to create eco-friendly and biodegradable disposable tableware, a high-volume single-use item. Cellulosic pulp from the tegument of mango (cultivar Tommy Atkins) was successfully obtained by a semichemical pulping process to manufacture biodegradable plates. Refining times of 0, 5, 10, and 15 min were tested, and it was observed that a refining time of 10 min yielded a notably stronger material of 2.15 km in breaking length. Moreover, when the mango fibers were blended with pine fibers in a 70:30 (mango/pine) ratio, the material’s porosity significantly improved from 0 to 0.60 s/100 cc air. Alkyl ketene dimer at 1.5% was incorporated to impart water-resistant properties, changing the contact angle drop test from 0 to >120°. The biodegradation test indicated that the samples achieved a significant biodegradation level of 62.14 and 67.65 after 182 days of testing. The results demonstrated that the tegument from mango agro-industrial waste has the potential to be a source of cellulosic pulp to produce biodegradable and disposable tableware, contributing to the decrease in the use of conventional plastics.

## 1. Introduction

Conventional plastics such as polyethylene (PE), polystyrene (PS), polypropylene (PP), and polyethylene terephthalate (PET) have been extensively utilized in the manufacturing of disposable tableware, including plates, bowls, cutlery, coffee cups, and other items. Research on the global waste classification reported that take-out consumer items corresponded to 50–88% and were the most significant share across all aquatic environments (open waters, deep seafloor, nearshore seafloor, nearshore waters, shoreline, river waters, and river bed). This percentage mainly comprised take-out consumer items, like plastic bags and wrappers, food containers and cutlery, and bottles and cans [1]. Undoubtedly, improper disposal of conventional plastics has negatively impacted the ecological system, leading to severe consequences for marine fauna [2]. Consequently, it is imperative to study new sources of raw materials to produce biodegradable disposable articles to promote a decrease in the generation of non-biodegradable wastes. One alternative to produce disposable tableware is cellulosic pulp, traditionally obtained from wood. Nowadays, agro-industrial wastes are explored as new sources of raw materials to extract cellulose fibers [3]. For example, cellulosic pulp from natural fibers is a non-conventional source that offers a biodegradable, renewable, often sustainably harvested, and non-toxic option.

In addition, the properties of these materials make them well-suited to the development of tableware, as they possess characteristics such as low weight, high tensile strength and Young’s modulus, tear-abrasion resistance, and heat resistance, among others [4]; different authors have developed biodegradable cups from sugarcane and bamboo fibers treated with water, which exhibited low heavy metal content, oil-grease resistance, shape retention at high temperatures, and an effective barrier against water vapor [5,6]. Saini et al. [7] successfully manufactured disposable rice straw bowls, producing biodegradable and food-safe products with superior mechanical properties, water and grease resistance, and energy-economic efficiency. The combination of natural fibers improves the mechanical properties. However, the additives that impart barrier properties could negatively impact the biodegradability.

Another potential source of natural fibers for developing tableware is mango (*Mangifera indica* L.) seed, a significant by-product of mango industrialization. Mango seed constitutes approximately 9–40% of the whole fruit [8]. The tegument is the fibrous exterior layer of the mango seed that protects the kernel and maintains its integrity; it is composed of lignocellulosic material (cellulose, hemicellulose, and lignin). Currently, some works have been reported on the chemical treatments of the tegument to obtain cellulosic fibers for composite development. Cordeiro et al. [9] evaluated the addition of tegument nanocellulose whiskers as a reinforcing agent in elaborating materials with thermoplastic starch from mango kernels. The resulting extruded material demonstrated favorable mechanical properties. In another study, researchers explored the formulation of a rigid packaging material using PLA (poly lactic acid) combined with 10% and 20% ground mango tegument. Adding 20% of tegument improved the mechanical and barrier properties significantly, compared to the control sample (PLA 100%), highlighting the potential of tegument as an effective reinforcing agent [10]. Furthermore, cellulosic pulp from the tegument of mango obtained by chemical treatments possesses properties suitable for incorporation into tableware formulations, such as crystallinity and thermal resistance [11]. In this work, we present a two-purpose research in which valorization of mango agro-industrial waste is performed by transforming the mango tegument into cellulosic pulp to produce disposable tableware. This high-demand biodegradable tableware should positively impact the replacement non-biodegradable material disposed of in the sanitary landfill.

The present study aimed to contribute to the decrease in the use of conventional plastics for disposable tableware by transforming the mango tegument into a biodegradable disposable plate with acceptable physical and mechanical properties through the semichemical pulping process using sodium hydroxide (NaOH) and further formulation.

## 2. Materials and Methods

### 2.1. Materials

Fruxo provided Mango (Tommy Atkins) agro-industrial waste from Tepic, Nayarit, México. Due to the peak mango season from April to August and the need for waste material throughout the year for this and other research purposes, it was convenient to freeze the waste at −20 °C until use. Nonetheless, this stage is not recommended for industrial use. The tegument of the mango was separated from the kernel before the application of the semichemical process. NaOH, hydrogen peroxide (H_2_O_2_), sulfuric acid (H_2_SO_4_), acetone (C_3_H_6_O), toluene (C_6_H_5_CH_3_), and heptane (C_7_H_16_) were purchased from Fagalab^®^, Mocorito, Sinaloa, México; acetic acid (CH_3_COOH) from J.T. Baker CTR Scientific, Monterrey, N.L., México; sodium chlorite (NaClO_2_) from Golden Bell Proquisur, CDMX, México; alkyl ketene dimer (AKD) dispersion was obtained from Ecofy Chem, Guangdong, China (tradename EC-PA01) and used without modifications (more information is presented in Table S1, Supplementary Material); cellulosic pulp from pine was donated by the Departamento de Madera, Celulosa y Papel, Guadalajara, Jalisco, México; disposable commercial plates were acquired in a local market (wheat straw, Wecare^®^, Zapopan, Jalisco, México).

### 2.2. Characterization of Fibers

#### Cellular Structure of Tegument of Mango and Physical Analysis of Fibers

The fibers characterization was carried out according to TAPPI 259 om-21. For the individual measurement of the fibers (diameter d, length L, lumen l, and wall thickness w), slivers of the tegument of mango were macerated in glass assay tubes with a solution of Franklin (1:2 glacial CH_3_COOH and 30% H_2_O_2_) to not alter their structure, in a water bath at controlled temperature 60 ± 5 °C until the fibers were dissociated, soft and whitish (48 h). Next, the fibers were washed with water until the reagents were eliminated. Subsequently, the fibers were put in slides, stained with safranin, dispersed with water, and covered with coverslips. Finally, the properties of the fibers were determined by an optical microscope (Leica^®^ DM500, Wetzlar, Germany) coupled with a digital camera “Nikon Coolpix 5400” (Nikon Corp., Tokyo, Japan) and analyzed with the ImageJ software version 1.53a (30 random measurements). The Runkel ratio (2 w/l), coefficient of flexibility (l/d), and slenderness ratio (L/d) were calculated based on the measured data. For the cellular structure, a piece of mango tegument was submerged in boiling water for two hours to soften the material. When the piece was soft, a 2 cm specimen was cut and placed in boiling water for an additional hour to prevent changes in its structure. Cuts of 20 µm of thickness from a transversal and longitudinal anatomical face were made with a sliding microtome (Microtome American Optical model 860, Vernon Hills, IL, USA) [12]. Scanning Electron Microscopy (SEM) analysis was carried out to evaluate the microstructure of the tegument of the mango using a MIRA 3 MULU (TESCAN, Brno, Czech Republic) instrument. The samples were coated with gold for 40 s using an acceleration voltage of 10 keV according to previously established protocols [13].

### 2.3. Semichemical Pulping Process

The semichemical pulping process was carried out by immersion of the tegument of the mango in a 19% (*w*/*v*) NaOH solution. The hydromodule was 1:9 (w:v) for one hour in a high-pressure digester at 165 °C. Once the process was finished, the tegument was washed with water to remove the excess NaOH until pH 7. Next, the cooked teguments were defibrillated with a laboratory scale refiner (Sprout–Waldron M105A, Muncy, PA, USA) with a gap width of 2.54 mm, three times. Finally, the fibers were selected using a diaphragm screen (Lorentzen Wettre, Stockholm, Sweden) with an aperture size of 0.15 mm. The cellulosic pulp was centrifuged and stored at 8 °C until use. Since NaOH was used in the semichemical process, the produced pulp will be named alkali cellulosic pulp in the present work, and the process will be named alkali treatment.

### 2.4. Chemical Analysis

The untreated fibers and the cellulosic pulp were analyzed according to previously established protocols in the literature regarding extractives, lignin, and holocellulose content [14,15].

The FTIR spectra of the cellulosic pulps were analyzed using a Thermo Fisher Scientific Model Nicolet iS50 FT-IR (Madison, WI, USA) operated in attenuated total reflectance (ATR) mode with a diamond crystal cell. Scans were collected from 4000 to 400 cm^−1^ to obtain the spectra (absorbance mode, 64 scans, and a resolution of 4 cm^−1^) [9,16].

### 2.5. Paper Sheets and Physical–Mechanical Properties

Paper sheets were prepared to determine the optimal refining time of the cellulosic pulp produced from the mango tegument. The pulp was refined in a Jokro mill (P.J. & Söhne Gmbh, Düren, NW, Germany) according to ISO 5264/3 at 0, 5, 10, and 15 min. The refined pulps were analyzed with Schopper Riegler (° SR) equipment to evaluate the refining degree based on the ISO 5267/1 method. Afterward, paper sheets of 100 GSM were prepared with the pulp using a laboratory hand-sheet former Rapid Köthen (Testing Machines Inc, New York, NY, USA) according to ISO 5269/2. The physical–mechanical properties of samples were analyzed according to TAPPI Standards: grammage (T-410 om-08), thickness (T-411 om-97), apparent density (T-220 sp-01), porosity (T-460 om-96), folding endurance (T-423 om-07), breaking length (T-494 om-01), and tear index consistency (T-414 om-12).

Once the refining time was selected (10 min), circular paper sheets (d = 16 cm) were made with blends of mango and pine cellulosic pulps in different formulations of mango/pine (100:0, 70:30, and 50:50), and their physical–mechanical properties were evaluated. The formulation with the best properties was selected for the circles that make the disposable plates.

### 2.6. Plate Development and Physical–Mechanical Properties

**Figure 1:**
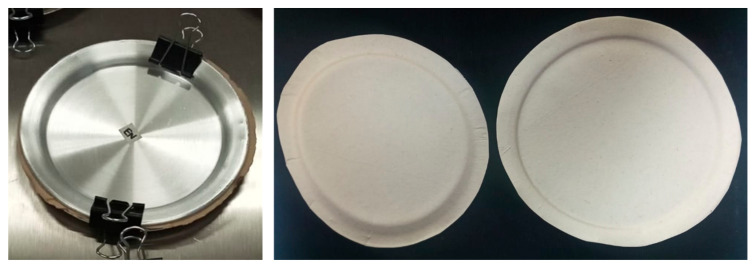
Plate-making process.

For the development of disposable plates, thicker circular paper sheets of 300 GSM were prepared with the formulations 100:0 and 70:30 of mango/pine; AKD was added at 1.5% as an additive to improve the hydrophobicity of the material in the suspension (internal sizing). Once the circular sheet was formed, the materials were molded between two stainless steel plates (d = 15 cm, Figure 1) and cured in an oven at 90 °C for 30 min. Afterward, the developed plates were dried at room temperature for 24 h. The physical–mechanical properties were analyzed, and the contact angle drop test was evaluated according to T-458 cm-04 [7]. Eight disks and plates were produced for each treatment.

### 2.7. Barrier Properties

The grease-oil resistance was determined according to TAPPI T559 cm-12. This method is also known as the TAPPI kit test, and the results are presented in the form of a kit number, indicating the level of resistance. Three replicates were evaluated for each disk of 300 GSM [17].

The water absorption capacity (Cobb_60_) of the disks of 300 GSM was assessed following the guidelines of TAPPI T 441 om-13. The samples were conditioned (23 ± 2 °C temperature and 50 ± 2% relative humidity) to determine the quantity of water absorbed per unit area in 60 s. One side of each disk was uniformly wet for that specific period. The Cobb_60_ value, which indicates the weight difference per unit area, was then calculated. Three samples were conducted for each disk (mango, mango/pine, and commercial), and the results were averaged [18].

The Water Vapor Transmission Rate (WVTR) was determined using the TAPPI T 448 om-21 and TAPPI T 464 om-22 standards. The samples of 300 GSM were conditioned at 21 °C and a relative humidity of 50% to simulate typical conditions, and at 41 °C, a relative humidity of 70% was used to simulate extreme conditions. Before the test, the weight of CaCl_2_ was measured, and all samples were mounted in stainless steel cells. Each sample was weighed every 24 h for 10 days. The WVTR of the samples was then calculated using Equation (1):

(1)  WVTR=k/permeation area

where k represents the slope of water gain by CaCl_2_ over time [19].

### 2.8. Overall Migration (OM) in Food Simulants

OM testing was conducted following the guidelines of the European Commission [20] to assess the total mass of compounds transferred from the disks of 300 GSM in food simulants. The food simulants used included a 10% ethanol solution to simulate aqueous foods, a 50% ethanol solution to simulate foods with lipophilic characteristics, and a 3% acetic acid solution to simulate acidic foods. Since the plates are intended for short-term contact with foods (<30 min at cool or warm conditions), the testing conditions were set at 40 °C for 30 min, representing the temperature and time typically encountered during brief food-plate contact [21]. Using a glass cell, the sample was exposed to the simulants under these specified conditions on one side. After the exposure, the simulant was evaporated, and the residue was weighed. The migrated materials were quantified as mg per dm^2^ of the food contact surface. All measurements were performed in three replicates.

### 2.9. Biodegradation Test

The biodegradation of material was evaluated according to ASTM D5988 and based on the work of Val-Félix et al. [22], with some modifications. The test assessed aerobic biodegradation in soilunder natural conditions using respirometers at 27 °C. Samples (one g of one mm^2^ pieces) were distributed between two equal layers ofsoil (50 g) into a glass Mason jar (480 mL) equipped with a CO_2_ and moisture capture filter. A plastic device with 30 mL of KOH solution (0.5 N) was used to trap the CO_2_ produced by mineralization. The captured CO_2_ was titrated with HCl (0.25 N) every week for 182 days. Five replicates were analyzed for the soil (blank), mango paper, mango paper with AKD, cellulosic pine pulp (positive control), and low-density polyethylene (negative control). The production of CO_2_ (mg) was determined by the difference between CO_2_ produced by the test materials and that produced by the test blank. The biodegradation (%) was established as the ratio between the net CO_2_ produced by the samples and the theoretical CO_2_ production (ThCO_2_) based on the total organic carbon (TOC). Biodegradation (%) was plotted as a function of incubation time (days).

### 2.10. Statistical Analysis

Data were analyzed by one-way ANOVA using NCSS statistical software (NCSS, 2021). Differences among means were compared using the Tukey–Kramer test (α = 0.05). All results are reported as the mean ± standard deviation.

## 3. Results and Discussion

### 3.1. Physical and Morphological Analysis of the Fibers

The physical characteristics of fibers play a crucial role in determining their suitability for various applications in the paper and cardboard industries. Table 1 presents the morphological properties of fibers from the tegument of mango, which are essential for evaluating their potential applications. The fiber length was 1.08 mm, while the fiber diameter was 19.1 µm, with a wall thickness of 6.0 µm. These values are comparable to other short fibers derived from various agro-industrial waste sources, such as rapeseed straw, maize stalk, and sunflower stalk [23]. However, the observed high fiber wall thickness can negatively impact multiple properties, such as folding endurance, since the paper sheet may not be well-formed. According to the coefficients obtained, the Runkel ratio indicates that the drying process of the paper will be affected by the thickness of the cell wall. Regarding the flexibility coefficient, the sheets will present low flexibility due to the reduced size of the lumen relative to the diameter of the fiber. In this case, the fibers will collapse somewhat due to the lack of contact surface [24]. The ratios suggest that the sheet formation with the fibers of the tegument might exhibit unfavorable properties. Nonetheless, the development of the plate does not need a high flexibility coefficient or extensive folding endurance because the material is for single-use applications and does not require repeated opening and reclosure [25]. The quality of the plates could be improved by mixing the mango fibers with a fiber lumen width higher than the fiber wall thickness.

### 3.2. Chemical Composition and Structural Analysis of the Fibers

The chemical composition of the untreated fibers and alkali cellulosic pulp is shown in Table 2. After the alkali treatment, there was a significant decrease (*p* < 0.05) in both organic and water extractives. These changes can be attributed to the effective removal of impurities and surface cleaning due to the NaOH reactions with lipophilic substances (resins, waxes, sterols, fats, or fatty acids) and polar fractions (tannins, gums, or sugars) [15]. The alkali cellulosic pulp exhibited a significantly higher (*p* < 0.05) holocellulose content of 84.7% compared to the untreated fibers, which had 67.2%. This difference can be attributed to the rise in cellulose content and the removal of amorphous constituents such as hemicelluloses and extractives [26]. The increased holocellulose content offers several advantages in the development of the material, including enhanced strength and biocompatibility [27]. No significant differences (*p* > 0.05) in lignin content were observed following the alkali treatment because hemicelluloses were removed more than lignin, primarily due to their more readily solubilized structure [11]. However, it is noteworthy that the lignin content in the studied material is lower than that of other residues, such as garlic and onion [28]. According to findings from Lorenzo-Santiago and Rendón-Villalobos [29], the lignin content in the alkali-treated mango tegument was reported to be 0.64%, which is much lower than our findings of 14.65%. This difference may be attributed to the additional treatments not included in our investigation. These treatments include bleaching and acid hydrolysis. Henrique et al. [30] found that the lignin content in untreated mango fibers was 23.85% higher than our results of 15.41%. The alkali treatment resulted in a cellulosic pulp characterized by reduced extractives and elevated holocellulose content.

Regarding the high volume of the NaOH solution compared with the tegument weight (hydromodule 1:9, w:v), these are conditions for batch-type digesters, like the one used in the present work for obtaining enough impregnation of the mango’s tegument and the fibers separation. However, at the industrial level, the use of continuous-type digesters is more efficient and complies with the same purpose at a lower pulp to alkali solution hydromodule (1:4, w:v). On the other hand, it is well known that the processes for producing cellulosic pulp generate undesirable contaminants. However, nowadays, most of the industrial processes have the regeneration and recirculation of the produced liquors already considered. Therefore, their application to the non-conventional agro-industrial wastes as starting materials diminishes the environmental impact while attempting to reduce the impact of these wastes in the area surrounding the mango fruit industry location.

**Figure 2:**
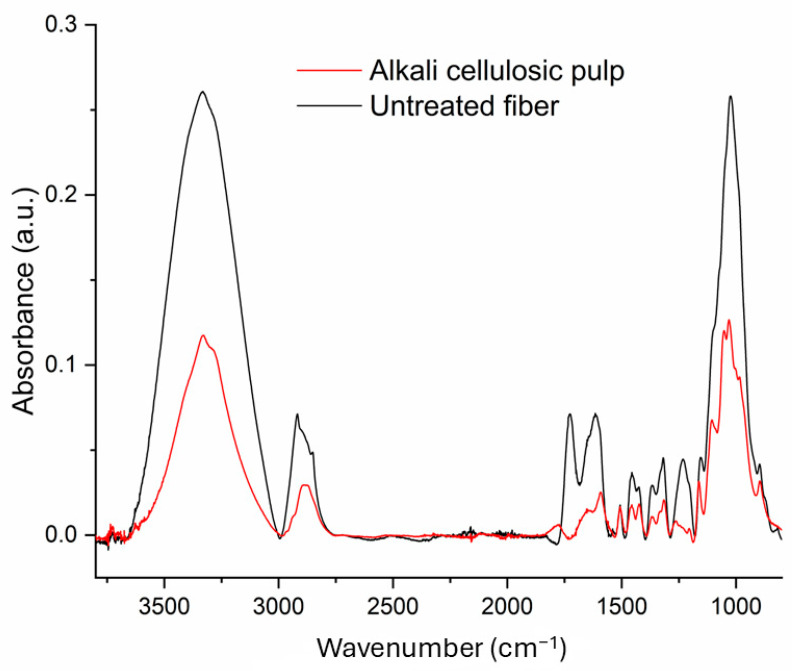
FTIR spectra of untreated fibers and alkali mango cellulosic pulp.

FTIR spectroscopy is a valuable technique for examining the structure of constituents and detecting chemical changes in lignocellulosic materials during treatments. Figure 2 shows the FTIR spectra of untreated fibers and alkali-treated cellulosic pulp. The broad band observed in the range of 3000 to 3600 cm^−1^ in untreated fibers was attributed to the stretching vibrations of hydroxyl groups in cellulose. However, after the alkali treatment, a remarkable decrease in the intensity of this band was observed. This reduction indicated a significant decrease in the hydroxyl group content, which can be attributed to the removal of amorphous cellulose and hemicelluloses. As a result, the surface of the material becomes more hydrophobic [31]. In addition, the peak observed at 1730 cm^−1^, which is attributed to the carbonyl group (C=O) of the acetyl group in hemicellulose, disappeared after the removal of hemicelluloses following alkali treatment [32]. The signal at 1237 cm^−1^ indicates that the stretching of C-O bonds in the acetyl groups of hemicellulose diminished after obtaining alkali cellulosic pulp. Similarly, the peak at 1030 cm^−1^, indicative of the stretching of C-O bonds in hemicellulose, showed a significant reduction in intensity [33]. The small signal at 1506 cm^−1^, attributed to the lignin’s presence [32], did not show variation after the alkali treatment. This behavior is consistent with the similar level of lignin found in the untreated fibers and alkali cellulosic pulp.

### 3.3. Scanning Electronic Microscopy (SEM)

**Figure 3:**
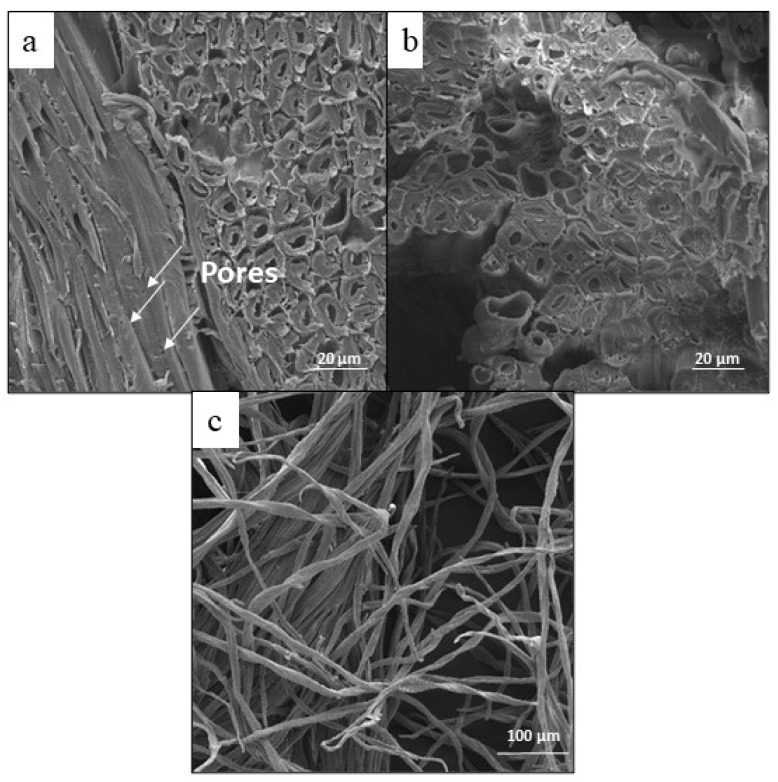
SEM images of tegument fibers of mango (**a**) longitudinal section on the left side (boiling water), (**b**) transversal section (boiling water), and (**c**) fibers from the tegument of mango (Franklin’s method).

SEM micrographs of the untreated tegument fibers are presented in Figure 3. The tegument of the mango exhibits a distinctive compact fibrous structure, which shows a level of structural complexity. Additionally, it features tiny pores that are distributed throughout its surface. The structure comprises an epidermis, a cellular inner region, and the lumen, similar to wheat straw fibers [34].

**Figure 4:**
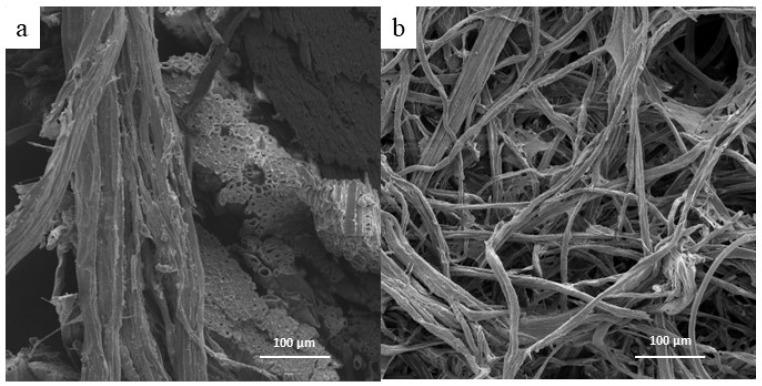
SEM images of tegument fibers of mango (**a**) transversal and longitudinal distribution (boiling water), and (**b**) alkali cellulosic pulp.

Figure 4 shows the fibers before (a) and after the alkali treatment (b). This treatment removed hemicellulose and other impurities present on the fiber surface. It is essential to note that the treatment caused significant modifications to the complex structure within the fibers of the tegument, resulting in their separation. Cordeiro et al. [9] also observed the removal of impurities from the fiber surface of the tegument of mango through alkali treatment and hydrogen peroxide, leading to the production of bleached cellulosic pulp. In the present work, the fiber arrangement on the tegument of mango changed as indicated by the FTIR results, leading to the release of fibers.

### 3.4. Physical and Mechanical Properties of Paper Sheets

Refining is the mechanical process of separating, fibrillating, cutting, and hydrating fibers to improve strength and sheet formation and promote bonding with other fibers. The fibers are modified due to internal fibrillation, external fibrillation, or fiber shortening [35]. Table 3 presents the effect of refining time on beating level, grammage, thickness, density, folding endurance, tear index, and breaking length. The sheets’ best physical and mechanical properties were achieved in a refining of 10 min without excessive energy consumption. The cellular morphology and fiber length affect the refining time. In the case of mango cellulosic fibers, which are relatively short, a lower refining time is required compared to longer fibers such as pine wood (30 min) [36]. Refining cellulosic pulp for 10 min reduced the thickness of the sheets and increased their density. These results can be attributed to enhanced fiber-fiber interaction, resulting in higher-density sheets. In addition, the tear index and breaking length exhibited significant enhancements corresponding to the increase in refining times, similar to the findings observed in Tunisian Alfa stem fibers [37]. The tear index increased at different refining times due to the interaction between fibers through hydrogen bonds [6].

On the other hand, the lower folding endurance values remained consistent across various refining times, ranging from 1.00 to 1.17. A thick cell wall affects the folding endurance of the sheet, resulting in a bulky material with a coarse surface and a high void volume [38]. The cell wall thickness of the fiber of tegument was 6.03 µm, which is relatively thick compared with the fiber cell walls of *Acacia* (2.51 µm) that can form sheets with high folding endurance [39]. Although a round and flat plate does not require high folding endurance, a potential strategy for enhancing this property in mango sheets is incorporating a proportion of long fibers during the beating step. Long fibers possess a robust network structure that imparts high resistance to folding. Combining such fibers into the sheet can result in a higher-density sheet due to the strong network formed.

Table 4 presents the three formulations of mango and pine cellulosic pulps (up to 50% concentration) to enhance the mechanical properties. The 70:30 formulation demonstrated superior mechanical properties in terms of tear index, folding endurance, and breaking length compared with the formulation 100:0. These features can be attributed to the synergistic interactions between the long and short fibers present on the surface [7]. Incorporating cellulosic pulp pine improved the mechanical properties due to individual strength, and the inter-bonding of long fibers was higher than that of short fibers. The resulting network exhibited a notable presence of hydrogen bonds that efficiently bonded with the short fibers, filling the void spaces [7]. Measuring the porosity of mango sheets (100:0) was not feasible due to the air flux freely passing from one side to the other without resistance. The addition of pine fibers significantly improved the air resistance of the samples, with the 50:50 formulation demonstrating the highest level of air resistance. This performance can be attributed to the interaction between the long and short fibers, which generated denser networks and reduced the pore size, consequently decreasing air transport [40]. We selected formulation 70:30 for the development of plates due to similar values in mechanical properties and a high proportion of mango cellulosic pulp.

Water resistance is an important parameter when developing tableware using natural fibers. The hydrophilicity of natural fibers can be effectively modified by incorporating chemical substances like AKD. This additive alters the hydrophilic nature of natural fibers into a hydrophobic surface by reacting with cellulose hydroxyl groups to give a β-keto ester bond, which increases hydrophobicity. Table 5 shows the physical and mechanical properties of formulated sheets added with 1.5% (*w*/*w*) AKD. The contact angle drop test for the two formulations was >120°, indicating that the materials were water-resistant and could be used with intermediate-moisture foods [5]. The mechanical properties of folding endurance and breaking length exhibited improvement through the interaction with long pine fibers in formulation 70:30 [6].

The inclusion of AKD transformed the sheets’ chemical surface from hydrophilic to hydrophobic by introducing hydrophobic functional groups to the cellulose. Structurally, the lactone rings in AKD react with the hydroxyl groups, forming β-keto esters and creating a hydrophobic film [41]. Our results are similar to those of tableware (cups) designed with sugarcane bagasse: bamboo (70:30) [6]. As the mix showed low water resistance, (0.5–1.0) % of AKD was added to give a cup with a water contact angle of 127°, slightly higher than the results of our plates. In another work, plates made with rice straws showed a water contact angle as low as 10.2°, and after coating with AKD at 12.2–30.3 g/m^2^, the contact angle increased to (94.9–119.2)° [7]. Slightly lower than our results with mango seed plates. These cases confirm the low water resistance of cellulosic pulp obtained from non-wood sources, a drawback that is controlled by the addition of AKD.

### 3.5. Barrier Properties of the Tableware Plate

Several properties should be tested in single-use tableware made from natural fibers and designed to be used in contact with food. It is essential to understand the behavior of the plates when they come into contact with fats/oils and water (in liquid and vapor form) since their principal function is to contain foods with diverse compositions. Table 6 presents the barrier properties of the plates: water resistance, grease resistance, and WVTR. The results were compared with those of a commercial tableware plate made of wheat straw.

As for grease resistance, the kit scale ranges from 0 to 12, with 12 denoting the highest level of resistance and 0 indicating no resistance. All the formulated tableware plates showed no grease resistance, as shown by the kit rating value of 0. In contrast, the commercial plate demonstrated moderate resistance with a kit rating of 6. The lack of grease resistance in our containers can be attributed to the high porosity of the material and the high AKD capacity to interact with oil [42]. Most commercial plates contain kaolin as a filler, which blocks the pores and delays grease penetration. As the mango/pine samples (100:0 and 70:30) formulations did not include this filler, its addition must be studied to improve their properties. However, as reported in this work, our plates still have the application to contain dry food like dry snacks or dried fruits, which are intended to be in contact with the plate for short times.

The Cobb_60_ value was used to determine the water absorption capacity of the samples, representing the mass of water absorbed in a specific area for 60 s. It was observed that the mango/pine samples (100:0 and 70:30) displayed lower water resistance than the commercial ones because they absorbed a relatively higher amount of water. The addition of AKD significantly increased surface hydrophobicity, attributed to the interaction between alkyl and cellulose hydroxyl groups. While the Cobb_60_ value in the mango/pine samples was higher than that of the commercial one, when compared with other materials coated with chitosan or cellulose nanofibers, these still demonstrate superior resistance to water absorption [40,43].

The WVTR values for the samples were from130 to 158 g/(m^2^ day) under typical testing conditions and from 792 to 1093 g/(m^2^ day) under extreme conditions. Under extreme testing conditions, the WVTR increased nearly seven times due to high water vapor flow. Nevertheless, the samples exhibited no surface cracks under both typical and extreme conditions, demonstrating that they could be stable even under high temperature and humidity conditions. Paper or paperboard’s water vapor transport mechanism involves diffusion through empty voids in the fiber structure. However, in samples treated with AKD, their alkyl groups and hydrophobic chains disrupted the water vapor absorption and, consequently, its diffusion [40,43].

### 3.6. Overall Migration (OM)

Table 7 shows the OM results for formulated materials and commercial samples in aqueous, acidic, and lipophilic food simulants. In the case of the aqueous food simulant (10% ethanol), a 30 min exposure at 40 °C yielded negative OM values ranging from −13.36 to −4.98 mg/dm^2^ attributed to the absorption of material of the simulant due to the content of ethanol. The formulation 100:0 demonstrated the highest absorption value, which can be explained by its porous structure that allows the simulant to permeate the plate more readily. Concerning the lipophilic food simulant (50% ethanol), the materials also absorbed liquid, resulting in negative values ranging from −14.14 to −1.96 mg/dm^2^. Similarly to the aqueous food simulant findings, the 100:0 formulation showed the highest absorption value, attributed to the higher ethanol content in the simulant that facilitated the transfer through the porous structure of the plate [44]. On the contrary, the OM in acidic food simulant was higher than the established limit by the European Commission (≤10 mg/dm^2^) for both the commercial and the developed materials. The contact of the samples with 3% acetic acid resulted in the random scission of the cellulose chains, forming carbohydrate molecules that underwent oxidation and promoted hydrolysis [45]. These products were released into the simulant, contributing to the OM value. According to the established limit for the European Commission, the evaluated materials exhibited a higher migration in acidic food simulant, failing to comply with the regulation. However, this regulation was established to test materials mainly made of plastics and does not represent contact with paper or paperboard. It appears that the testing conditions exaggerated the actual contact between solid food and the paper plates. Moreover, the presence of AKD was insufficient to seal the porous surface or the fibers and prevent the diffusion of ethanol or the hydrolysis of the cellulose chains. Tanpichai et al. [46] applied several layers of chitosan coating on cellulose-based paper, decreasing the absorption of liquids and reducing OM. Future work in our research is to find a bio-based and biodegradable coating material that effectively fills the pores and cavities of the plates and prevents the release of products of the hydrolysis of the fibers.

### 3.7. Biodegradation

**Figure 5:**
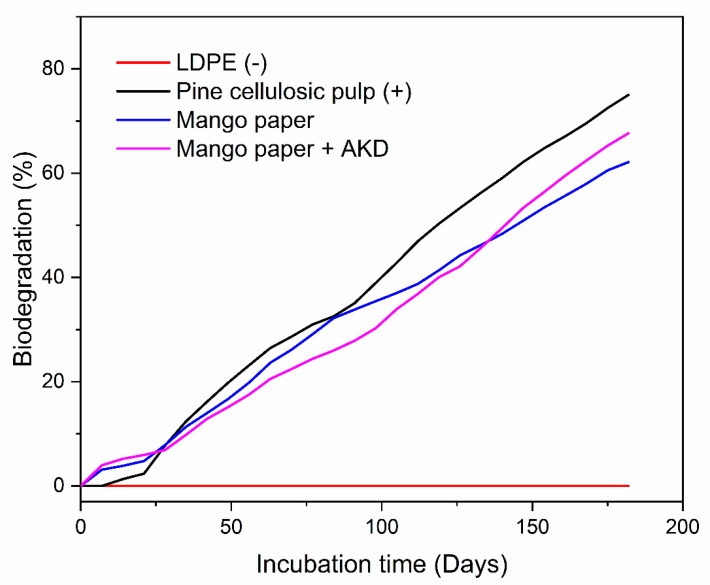
Biodegradation curves of pine cellulosic pulp (positive control), LDPE (negative control), mango paper, and mango paper + AKD during 182 days under controlled conditions (Soil: pH 7.0, humidity 50% and temperature 27 ± 1 °C).

Figure 5 presents the biodegradation curves of the studied materials in a controlled soil aerobic biodegradation test, comparing them with positive and negative controls. The biodegradation percentage of positive control (pine cellulose pulp) was 75.03 ± 0.70% at 182 days. According to ASTM D5988, the biodegradation run was considered valid because the positive control achieved a biodegradation higher than 70% after six months. Concerning the samples, the biodegradation percentage during 182 days of incubation for mango paper was 62.14 ± 0.87%, whereas for mango paper + AKD, it was 67.65 ± 0.92%. Biodegradation occurred in the samples at different rates since this process can be influenced by various factors such as cellulose molecular weight, crystallinity, and hydrophilicity [47]. The variations in biodegradation percentage between pine cellulosic pulp and mango paper can be attributed to differences in their lignin content. The industrial bleaching process applied to pine cellulosic pulp removed lignin, facilitating enhanced microbial access to cellulose during composting. In contrast, in mango paper, the presence of lignin hindered biodegradation, resulting in a reduced breakdown of cellulose fibers [48,49]. At the end of the assay, the biodegradation percentage of mango paper + AKD was higher than that of mango paper; it could be attributed to the high humidity (50%) and alkali pH (8.65), which influenced AKD hydrolysis [50]. The void spaces (free volume) could improve the enzymatic biodegradation rate. Togo and Hagihara [51] found a direct correlation between free volume and biodegradation rate with films developed with amorphous poly lactic acid. In the present work, the biodegradation curve showed two distinct stages: the lag phase (first 21 days of incubation), in which microorganisms adapt to the substrate and are characterized by low production of CO_2_, and the log phase (from 22 to 182 days) corresponds to biodegradation, in which the microorganisms obtain energy for their metabolic reactions with a significant increase in CO_2_ production. The plateau stage was not observed in the curve before 182 days of incubation.

## 4. Conclusions

Cellulosic pulp from the tegument of mango seed was obtained through a semichemical pulping process for manufacturing a disposable tableware plate. The mango tegument fibers were short, and the paper sheet had high porosity and low breaking length. Mixing mango fibers with pine fibers improved the mechanical properties of the plate, resulting in low porosity and high breaking length. Furthermore, adding AKD conferred hydrophobicity to the surface of the plate. However, AKD was insufficient to prevent the diffusion of liquid food simulants into the fibers during the OM study. The disposable plate passed with the biodegradability test, demonstrating the potential of mango agro-industrial waste as a source for developing tableware items that can reduce the consumption of disposable plastic items.

## Figures and Tables

**Table 1:** Morphological characteristics of the tegument fibers of mango seed.

Parameter	Value
Fiber length (mm) (L)	1.08 ± 0.18
Fiber diameter (µm) (d)	19.10 ± 2.96
Fiber lumen width (µm) (l)	6.67 ± 1.06
Fiber wall thickness (µm) (w)	6.03 ± 2.23
Runkel ratio (2 w/l)	2.60 ± 1.46
Slenderness ratio (L/d)	57.31 ± 10.21
Flexibility coefficient ((l/d)100)	30.99 ± 8.38

The values are the mean of thirty replicates ± standard deviation.

**Table 2:** Chemical composition of untreated fibers and alkali mango cellulosic pulp.

Fibers	Water Extractives (%)	Organic Extractives (%)	Holocellulose Content (%)	Lignin Content (%)
Untreated	17.39 ± 0.55 ^b^	4.64 ± 1.02 ^b^	67.17 ± 1.17 ^a^	15.41 ± 2.69 ^a^
Alkali	0.83 ± 0.14 ^a^	0.04 ± 0.00 ^a^	84.71 ± 0.09 ^b^	14.65 ± 1.53 ^a^

Each value is the mean of two replicates ± standard deviation. Different letters in the same column correspond to statistical differences between alkali treatments and untreated fibers (*p* < 0.05).

**Table 3:** Physical and mechanical properties of paper sheets made of tegument of mango alkali-treated at different refining times.

Properties	Refining Time (min)			
	0	5	10	15
Beating level (°SR)	17	19	22	20
Grammage (GSM)	110.96 ± 3.04 ^a^	106.63 ± 1.27 ^a^	105.40 ± 0.72 ^a^	105.56 ± 0.77 ^a^
Thickness (mm)	0.32 ± 0.01 ^c^	0.29 ± 0.01 ^b^	0.27 ± 0.00 ^a^	0.29 ± 0.01 ^b^
Density (g/cm^3^)	0.34 ± 0.00 ^a^	0.37 ± 0.00 ^b^	0.39 ± 0.00 ^c^	0.41 ± 0.00 ^d^
Folding endurance	1.00 ± 0.00 ^a^	1.00 ± 0.00 ^a^	1.17 ± 0.41 ^a^	1.17 ± 0.41 ^a^
Tear index (mN.m^2^/g)	0.33 ± 0.01 ^a^	1.12 ± 0.18 ^b^	0.80 ± 0.25 ^b^	1.55 ± 0.58 ^b^
Breaking length (km)	1.48 ± 0.27 ^b^	1.16 ± 0.08 ^a^	2.15 ± 0.17 ^c^	2.40 ± 0.20 ^c^

Each value is the mean of at least three replicates ± standard deviation. Different letters in the same row correspond to statistical differences among distinct refining times (*p* < 0.05).

**Table 4:** Physical and mechanical properties of paper sheets made of formulations from mango/pine cellulosic pulps.

Properties	Mango/Pine		
	100:0	70:30	50:50
Grammage (GSM)	105.40 ± 0.72 ^a^	90.74 ± 0.50 ^a^	91.25 ± 0.87 ^a^
Thickness (mm)	0.27 ± 0.00 ^a^	0.19 ± 0.01 ^a^	0.20 ± 0.01 ^a^
Folding endurance	1.17 ± 0.41 ^a^	3.17 ± 0.41 ^b^	2.50 ± 0.84 ^b^
Tear index (mN.m^2^/g)	0.80 ± 0.25 ^b^	1.46 ± 0.18 ^b^	0.72 ± 0.27 ^a^
Breaking length (km)	2.15 ± 0.17 ^c^	5.23 ± 0.58 ^b^	6.73 ± 1.68 ^b^
Porosity (s/100 c.c. air)	ND	0.60 ± 0.17 ^a^	1.17 ± 0.27 ^b^

Each value is the mean of three replicates ± standard deviation. Different letters in the same row correspond to statistical differences among mango/pine proportions (*p* < 0.05). ND: Not Determined due to the elevated porosity.

**Table 5:** Physical and mechanical properties of paper plates made of formulations from mango/pine cellulosic pulps added with 1.5% AKD.

Properties	Mango/Pine	
	100:0	70:30
Grammage (GSM)	254.70 ± 3.19 ^a^	268.72 ± 0.50 ^b^
Thickness (mm)	0.64 ± 0.01 ^b^	0.59 ± 0.02 ^a^
Folding endurance	1.00 ± 0.00 ^a^	2.00 ± 0.00 ^b^
Tear index (mN.m^2^/g)	0.37 ± 0.16 ^a^	0.37 ± 0.02 ^a^
Breaking length (km)	2.75 ± 0.88 ^a^	5.13 ± 0.56 ^b^
Porosity (s/100 c.c. air)	0.71 ± 0.07 ^a^	1.63 ± 0.13 ^b^
Contact angle (°)	122.14 ± 1.95 ^a^	122.71 ± 2.13 ^a^

Each value is the mean of three replicates ± standard deviation. Different letters in the same row correspond to statistical differences between mango/pine proportions (*p* < 0.05).

**Table 6:** Barrier properties of tableware plates formulated with mango/pine pulps added with 1.5% AKD, as compared to commercial plates.

Barrier Properties	Commercial	Mango/Pine	
		100:0	70:30
Grease resistance (Kit rating)	6 ^b^	0 ^a^	0 ^a^
Cobb_60_ (g/m^2^)	21.91 ± 0.60 ^a^	29.83 ± 1.49 ^b^	32.21 ± 3.69 ^b^
WVTR (g/m^2^ day) (21 °C, 50% R.H.)	130.09 ± 5.06 ^a^	137.16 ± 6.49 ^a^	158.29 ± 7.47 ^b^
WVTR (g/m^2^ day) (41 °C, 70% R.H.)	792.15 ± 6.59 ^a^	1093.05 ± 10.12 ^b^	1070.59 ± 28.89 ^b^

Each value is the mean of three replicates ± standard deviation. Different letters in the same row correspond to statistical differences between mango/pine and commercial plates (*p* < 0.05).

**Table 7:** Overall migration of commercial plates and disks formulated with mango/pine pulps and 1.5% of AKD.

Food Simulant	Commercial	Mango/Pine	
		100:0	70:30
	mg/dm^2^		
Aqueous (10% *v*/*v* ethanol)	−5.76 ± 0.99 ^a^	−13.36 ± 3.12 ^b^	−4.98 ± 1.38 ^a^
Lipophilic (50% *v*/*v* ethanol)	−7.86 ± 0.39 ^b^	−14.14 ± 1.18 ^c^	−1.96 ± 0.39 ^a^
Acidic (3% *v*/*v* acetic acid)	16.77 ± 1.49 ^b^	12.71 ± 0.82 ^a^	10.74 ± 1.86 ^a^

Each value is the mean of three replicates ± standard deviation. Different letters in the same row correspond to statistical differences between mango/pine disks and commercial plates (*p* < 0.05).

## Data Availability

The data that support the findings of this study are available on request from the corresponding author. The data are not publicly available due to privacy restrictions.

## Supplementary Materials

The following supporting information can be downloaded at: https://www.mdpi.com/article/10.3390/polym17202757/s1, Table S1: Chemical reagents used for processing and analysis.

## Abbreviations

The following abbreviations are used in this manuscript:

°SR	° Schopper-Riegler
AKD	Alkyl Ketene Dymer
ASTM	American Society for Testing Materials
ATR	Attenuated Total Reflectance
FTIR	Fourier Transform Infrared Spectroscopy
GSM	Grams per Square Meter
OM	Overall Migration
PE	Polyethylene
PET	Polyethylene Terephthalate
PLA	Polylactic Acid
PP	Polypropylene
PS	Polystyrene
ThCO_2_	Theoretical CO_2_
TOC	Total Organic Carbon
WVTR	Water Vapor Transmission Rate
