# Supplementary material for: Development and Properties of Disposable Plates Made of Cellulosic Pulp from Mango Agro-Industrial Waste
Source: Polymers (Basel). 2025 Oct 15;17(20):2757. doi: 10.3390/polym17202757 (PMC12566691; doi:10.3390/polym17202757)
Supplement: Supplementary file 1 [file polymers-17-02757-s001.zip › polymers-3871603-supplementary.pdf]

polymers

MDPI

Article

Development and properties of disposable plates made of cellulosic pulp from mango agro-industrial waste.

Maribel García-Mahecha [1], Herlinda Soto-Valdez [1*], María Guadalupe Lomelí-Ramírez [2], Hilda Palacios-Juárez [2], José Anzaldo-Hernández [2], Tomás Jesús Madera-Santana [1], Citlali Colín-Chávez [3], Elizabeth Peralta [1], Rafael Auras [4], and Elizabeth Carvajal-Millan [5]

[1] Coordinación de Tecnología de Alimentos de Origen Vegetal, Centro de Investigación en Alimentación y Desarrollo, A.C. (CIAD), Hermosillo, Sonora, 83304, México; maribel.garciam@hotmail.com, hsoto@ciad.mx, eperalta@ciad.mx, madera@ciad.mx
[2] Departamento de Madera, Celulosa y Papel del Centro Universitario de Ciencias Exactas e Ingenierías, Universidad de Guadalajara, Km 15.5 Carretera Guadalajara-Nogales, Zapopan, Jalisco, 45220, México; maria.lramirez@academicos.udg.mx, hilda.palacios@academicos.udg.mx, jose.anzaldo@academicos.udg.mx
[3] Centro de Innovación y Desarrollo Agroalimentario de Michoacán, A.C. (CIDAM), Morelia, Michoacán, 58341, México; citlali.colin@ciad.mx
[4] School of Packaging, Michigan State University, East Lansing 48424, Michigan, USA; aurasraf@anr.msu.edu
[5] Coordinación de Tecnología de Alimentos de Origen Animal, Centro de Investigación para Alimentación y Desarrollo, A.C., (CIAD), Hermosillo, Sonora, 83304, México; ecarvajal@ciad.mx

* Correspondence: hsoto@ciad.mx; Tel.: (+526622892400)

1. Supplementary Material

Table S1. Chemical reagents used for processing and analysis.

Name	Chemical formula	Brand	Batch number
Sodium hydroxide	$NaOH$	Fagalab®	1116-H2231
Hydrogen peroxide	H_2O_2	Fagalab®	2617-P2303
Sulfuric acid	H_2SO_4	Fagalab®	1816-A2047
Acetone	C_3H_6O	Fagalab®	1722-A2011
Toluene	$C_6H_5CH_3$	Fagalab®	2510-T2367
Heptane	C_7H_{16}	Fagalab®	1716-H2402
Acetic acid	CH_3COOH	J. T. Baker Scientific	L43C53
Sodium chlorite	$NaClO_2$	Golden Bell Proquisur	2012697
Alkyl ketene dimer	$C_{28}H_{52}O_2$	Ecofy Chem	-
